# Case of James P. Vance

**Published:** 1903-12

**Authors:** M. J. Bleim

**Affiliations:** San Antonio


### Case of James P. Vance.

---

BY DR. M. J. BLEIM, SAN ANTONIO.

---

*Editor Texas Medical Journal:*

[As a complete history can be given, it has seemed advisable to report this case rather fully.]

Aged, 28 years; 5 feet, 8 inches in height; weight, 180 pounds; ruddy complexion and robust in appearance; family history good; both parents and two brothers living. Has always enjoyed good health; habits, usually good—never drank anything; never had syphilis. During last illness discovered a slight urethral discharge which was not examined for diplococci. Has resided in San Antonio for three and a half years, where he has been following the profession of court stenographer. Has been in the city all summer and enjoyed perfect health. For over two years has lived at a large, firstclass boarding house at 140 North street, known as "The Roosevelt." This house is a large, two-story brick, in perfect sanitary condition, and is situated about two blocks from Alamo Plaza. He occupied a southeast corner room on the second floor. No other cases in this house or neighborhood.

On Thursday afternoon, November 5, 1903, at 2 o'clock, Mr. Vance was taken with a moderate chill, followed by moderate fever and some headache. As he was making preparations to go to Fort Stockton on court business, and had obtained his permit from Dr. Tabor, he, notwithstanding his fever, went at 4 p. m. to the Southern Pacific depot to deposit his baggage for fumigation. On Friday morning, at 10 o'clock, he called at my office and stated the above facts. He said he was not feeling very sick, but as he was going Saturday morning to a place sixty miles from a railroad, he thought he might need some medicine. He had no headache or backache; no nausea; tongue slightly coated and small with red tip; temperature 101½°; pulse 106. I advised him to retire to his room and report in the evening. As he objected to quinine, I gave him Tr. Gelsemium and Methlene Blue. At 5 p. m. he reported, saying he felt about the same. As his temperature had dropped one degree, I though it would disappear by morning and he could take his trip, and so advised him.

Saturday morning I was summoned to his room. Had vomited the night before. Temperature 103°, pulse 106; eyes quite suffused; tongue same as before; no special pain in head and back, and none in extremities. At night, temperature 103.2°, pulse 100; skin hot and dry. I took urine, which, on examination, showed albumin abundantly.

Sunday morning, temperature 102.8°, pulse 94. Had spent very restless night; no more vomiting; great thirst; no pain; felt very bad. Sunday evening blood was taken by Dr. Lloyd who, on Monday morning, reported no plasmodium. His slides were examined by three others who concurred in the absence of plasmodium. Urine taken Monday morning showed increased presence of albumin. On Sunday evening temperature was 103.8°, and pulse 92. Monday morning found temperature 102°, pulse 86. Had sweat profusely in night, and was very weak. Some tenderness in stomach; eyes slightly tinged yellow; tongue more pronounced red tip and edges, and small. At noon he was removed to the screened room in the City Hospital and was seen by Dr. S. Burg, city physician, and many others thereafter; among these were Drs. Tabor and Richardson, Lloyd, Elmendorf, Withers, Assistant City Physician Clavin, Shropshire, Dr. Marvin L. Graves and his assistants, Drs. Allison and Greenwood, Taylor, Bell, C. A. R. Campbell and A. Graves, Sr.

On Monday, about 4 p. m., vomiting began with streaks of blood. The eyes were now distinctly yellow. By Tuesday morning the whole body had turned a bright lemon yellow color, and the eyes were a golden yellow. The vomit was by this time distinctly blackish in color, settling like coffee grounds. Vomiting continued more or less until death, becoming purely disintegrated blood (typical black vomit). Under the microscope blood cells and free hemoglobin were found. The blood was again examined on Tuesday by Dr. Lloyd, and also by Dr. M. L. Graves and assistants on Wednesday, in each case with negative results as to plasmodium.

During Tuesday and Wednesday retention of urine occurred, but no suppression. Albumin, one-third in volume on standing twenty-four hours. The urine never was suppressed; this I attribute largely to the fact that the patient retained a pint of normal salt solution in the colon every three hours. Toward the end the urine became horribly offensive.

During Monday the temperature fluctuated between 104° and 102°, and pulse in the 80s. Thereafter the temperature never fell below 97° in the rectum, and mostly ranged between 98° and 99½°. The patient never suffered a collapse and the lowest pulse was 68 Monday night. From Wednesday night the pulse gradually ascended to 90 and all day Thursday became more and more rapid, while the patient passed first on Wednesday into an apathy, gradually into stupor and, finally into profound coma, with rapid pulse and respiration. On Thursday night, at 1:30 a. m. (early Friday morning) a convulsion occurred and at 1:45 a. m. he breathed his last.

Fortunately, a compete autopsy was allowed with, in brief, the following findings: Body, a deep lemon yellow color; black fluid oozing from mouth, ecchymoses on back and posterior aspects of extremities. Liver slightly discolored on under surface from post-mortem changes. One-half ounce of bile in gall-bladder; weight, one pound, thirteen ounces (avoir.); sixe 7x10 inches; on incision was of a uniform orange yellow color, and tissues pliable; spleen slightly enlarged (3½ wide, 5 inches long, 2 inches thick; weight, 9 ounces); on incision of a deep purplish color and friable; stomach contained 3¼ ounces of "black vomit." Mucous lining of stomach and duodenum deeply ecchymosed and of blackish color. Intestines distented with gas and of greenish color. Kidneys slightly enlarged and greatly congested, with hemorrhages; right kidney hemorrhagic in part under capsule size of a silver quarter. Heart rather small and tissues moderately firm.

A distinctly offensive odor emanated from his person during the last two days, and also the characteristic troubled countenance was very evident.

A few brief remarks may be in order. Several typical symptoms were absent. The patient never suffered the excruciating pains in head and back. He went into the fever very slowly; although he had his chill Thursday at 2 p. m., he was not really very ill until Friday night. Then, again, he never suffered the collapses so often seen, consequently his temperature and pulse never sank, as they often do, to 96°, and 40 or 50 beats per minute, nor did he have complete suppression of urine. I have given my explanation for this above.

The salient diagnostic symptoms are as follows: The usual onset with chill and fever with no remission of any consequence until after seventy-two hours. The characteristic lack of correlation between pulse and temperature on the second and third days, when, starting with a temperature of 101½° and pulse 106, the temperature finally reached 103.8° with a pulse of 92. The suffused eyes, gliding into a golden yellow; the general jaundice, first decidedly noticeable on the fifth morning, and of characteristic lemon yellow color; the typical tongue and spongy gums; tenderness in epigastrium; pain in the calves at one time; the typical black vomit; high grade of albuminuria, developing (early) on second day; clinically careful percussion of liver and spleen showed no enlargement; repeated blood examinations showed absence of plasmodium. History of case showed no previous malarial infection. This fact, and absence of plasmodium, absolutely rule out pernicious malaria. After the autopsy, which was attended by fifty physicians, a few dissented from the almost unanimous verdict of yellow fever, and said it was "acute yellow atrophy of the liver." The sole basis for such a diagnosis was the light weight of the liver. In size it was not much diminished below the normal; certainly nothing like it would be in acute yellow atrophy. Furthermore, the extreme rarity of this disease—only a little over 200 ever reported (Strümpell)—and the clinical history of the case, together with the other decisive symptoms, all would rule out the possibility of "acute yellow atrophy."

In conclusion, it is necessary to state that the city bacteriologist and pathologist who saw the case on Tuesday, did not deem it necessary to take a drop of the patient's blood and make the crucial test of and examination for the plasmodium, consequently his adverse diagnosis of "pernicious malaria" carries no weight whatever. Of all the physicians who saw the patient during his illness only two dissented from the diagnosis of yellow fever. These two gentlemen have uniformly and persistently contradicted the diagnosis of yellow fever in every case reported in San Antonio. The intelligent members of the profession can form their own conclusions.

